# YAP and TAZ in epithelial stem cells: A sensor for cell polarity, mechanical forces and tissue damage

**DOI:** 10.1002/bies.201600037

**Published:** 2016-05-13

**Authors:** Ahmed Elbediwy, Zoé I. Vincent‐Mistiaen, Barry J. Thompson

**Affiliations:** ^1^ Epithelial Biology LaboratoryFrancis Crick InstituteLondonUK

**Keywords:** epithelial polarity, Hippo pathway, mechanosensing, mechanotransduction, TAZ, wound healing, YAP

## Abstract

The YAP/TAZ family of transcriptional co‐activators drives cell proliferation in epithelial tissues and cancers. Yet, how YAP and TAZ are physiologically regulated remains unclear. Here we review recent reports that YAP and TAZ act primarily as sensors of epithelial cell polarity, being inhibited when cells differentiate an apical membrane domain, and being activated when cells contact the extracellular matrix via their basal membrane domain. Apical signalling occurs via the canonical Crumbs/CRB‐Hippo/MST‐Warts/LATS kinase cascade to phosphorylate and inhibit YAP/TAZ. Basal signalling occurs via Integrins and Src family kinases to phosphorylate and activate YAP/TAZ. Thus, YAP/TAZ is localised to the nucleus in basal stem/progenitor cells and cytoplasm in differentiated squamous cells or columnar cells. In addition, other signals such as mechanical forces, tissue damage and possibly receptor tyrosine kinases (RTKs) can influence MST‐LATS or Src family kinase activity to modulate YAP/TAZ activity.

**Abbreviations**
ECM: extracellular matrix
EGF(R): epidermal growth factor (receptor)
RTK: receptor tyrosine kinase

## Introduction

Animal tissues, from *Drosophila* to humans, tend to harbour a population of stem cells that is responsible for maintaining the tissue through cell proliferation and differentiation of daughter cells 1, 2, 3, 4, 5. Stem cells can proliferate to maintain normal tissue homeostasis, but also increase their proliferation in response to mechanical stretching of the tissue or to tissue damage and consequent inflammation. For example, the normal growth of the skin from newborn to adulthood occurs through stretching of the tissue, which promotes proliferation of basal layer stem/progenitor cells. In addition, wounding or infection of the skin also triggers a proliferative response of basal layer cells to replace the damaged skin with new cells. How these events are orchestrated at the molecular level, and whether they become deregulated in human epithelial cancers, is still poorly understood.

Recent discoveries from *Drosophila* genetics identified the YAP/TAZ family of transcriptional co‐activators (the sole *Drosophila* homologue is called Yorkie) as being essential regulators of cell proliferation during development and in adult stem cells of the intestine 6, 7, 8, 9. *Drosophila* Yorkie drives transcription of pro‐proliferative target genes through interaction with the TEAD‐family DNA binding transcription factor Scalloped, as well as additional co‐factors MASK, WBP2 and Brahma 10, 11, 12, 13, 14, 15, 16. Importantly, Yorkie is regulated by the cell polarity machinery in epithelial cells, being activated upon loss of the apical polarity determinant Crumbs, or loss of the planar polarity determinant Fat 17, 18, 19, 20, 21, 22. There is also evidence for Yorkie acting as a sensor of mechanical forces during development, where it promotes cell proliferation in response to epithelial stretch forces acting on the cytoskeleton 23, 24. Furthermore, Yorkie activity is induced upon tissue damage to promote intestinal stem cell proliferation and tissue repair 7, 8, 9, 10.

**Figure 1:**
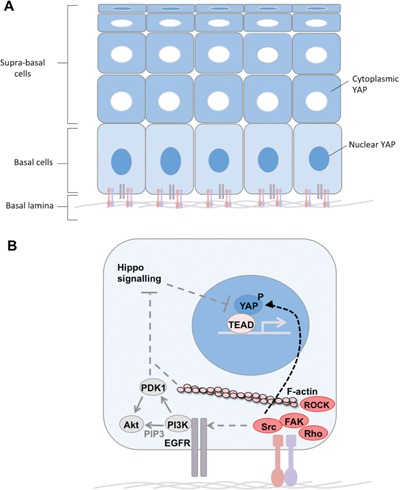
Basal signals promote nuclear YAP localisation. **A:** In stratified squamous epithelia, YAP/TAZ is nuclear in the basal cell layer which contacts the basal lamina ECM via Integrins. Supra basal cells lose contact with the basal lamina and thus experience reduced Integrin signalling and relocalisation of YAP/TAZ to the cytoplasm. One exception are the extremely flattened terminally differentiated cells, where YAP/TAZ can once again become nuclear, possibly due to mechanical stretching. **B:** Integrin‐Src‐FAK signalling synergises with EGFR‐PI3K signalling to promote nuclear localisation of YAP. Src can directly tyrosine‐phosphorylate YAP, but may also act indirectly to inhibit Hippo signalling, which inhibits YAP via serine/threonine phosphorylation to promote cytoplasmic retention. PI3K induces PIP3 lipid formation, which may help stabilise Integrin adhesions as well as inducing PDK1 and Akt activation. F‐actin, Rho and ROCK also generate actomyosin contractility to help stabilise Integrin adhesions and thus may contribute to Src activation.

**Figure 2:**
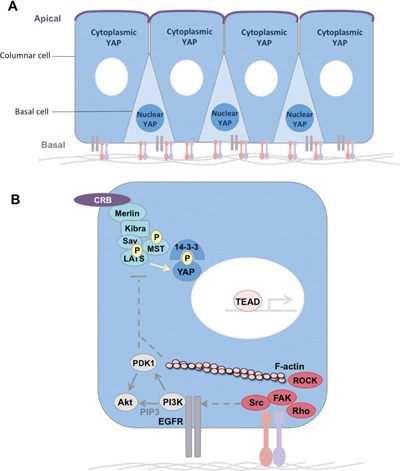
Apical signals inhibit nuclear YAP localisation. **A:** In columnar epithelia, YAP/TAZ is cytoplasmic in differentiated cells with an apical domain and nuclear in basal layer stem cells which lack an apical domain and contact the basal lamina ECM via Integrins. **B:** Crumbs‐Merlin‐Kibra‐Salvador‐MST‐LATS signalling (the canonical Hippo pathway) leads to phosphorylation of YAP/TAZ and retention in the cytoplasm (due to binding to 14‐3‐3 proteins) despite contact with the ECM. Thus, strong apical Hippo signalling is able to overcome basal Integrin signalling to maintain YAP/TAZ in the cytoplasm.

**Figure 3:**
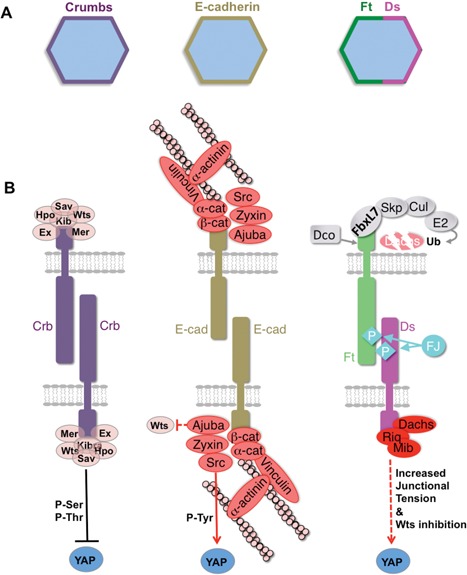
Regulation of YAP by Crumbs and Cadherin signalling. **A:** Crumbs and E‐cadherin distribute around the entire circumference of the epithelial cell's apical surface. In contrast, Fat and Dachsous cadherins planar polarise to opposite ends of the cell. **B:** Crumbs signals via canonical Merlin‐Ex‐Kibra‐Sav‐Hpo‐Warts/LATS signalling to inhibit YAP by direct ser/thr phosphorylation and cytoplasmic retention. E‐cadherin recruits Ajuba/Zyxin proteins, which may directly inhibit Warts/LATS kinases and Src family kinases, which tyrosine phosphorylate and activate YAP. Dachsous recruits the Dachs myosin, which increases junctional tension, as well as Riq and Mib, which may directly inhibit Warts/LATS kinases.

**Figure 4:**
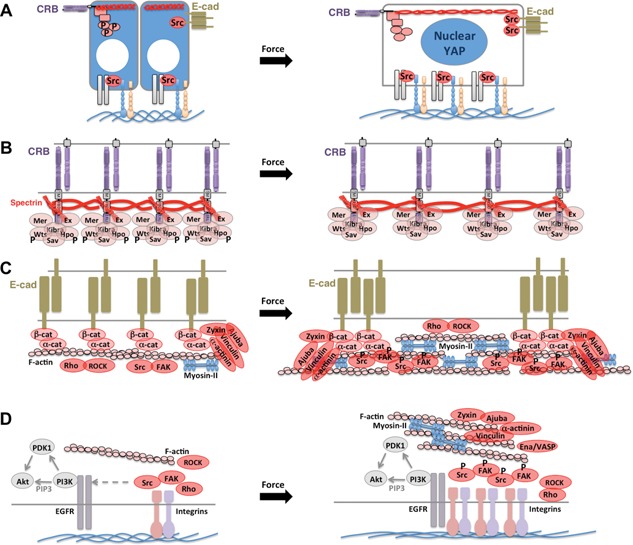
Models of mechano‐sensing that may control YAP localisation. **A:** Columnar epithelial cells exhibit cytoplasmic YAP at high density, but nuclear YAP at low density (which induces spreading out of cells). **B:** Model for inhibition of apical Crumbs‐Hippo signalling upon cellular stretching (due to de‐clustering of Crumbs complexes). **C:** Model for activation of Src at adherens junctions upon cellular stretching (due to induction of actomyosin contractility to resist stretching, clustering of adherens junctions, and recruitment of Ajuba/Zyxin family proteins as well as alpha‐actinin and Vinculin). **D:** Model for activation of basal Integrin‐Src signalling upon cellular stretching (due to formation of focal adhesions which cluster Integrins and recruit Ajuba/Zyxin, alpha‐actinin and Vinculin).

Here we review the molecular mechanisms responsible for regulation of Yorkie by cell polarity, force and damage in *Drosophila*. We then examine the regulation of YAP and TAZ in different mammalian epithelial tissues in vivo, which points to the existence of fundamentally conserved mechanisms between *Drosophila* and mammals. We also examine the regulation of YAP and TAZ during human epithelial cancer progression, where disruption of cell polarity, invasive migration, as well as damage and inflammation all appear to promote the action of YAP and TAZ in the nucleus. Our observations outline a unifying regulatory logic controlling YAP/TAZ co‐activators (summarised in Figs. 1, 2, 3, 4) and also suggest avenues for therapeutic intervention in inflammation and cancer. Finally, we are critical of results in cell culture that are unsupported by related findings in vivo.

### Yorkie as polarity‐sensor, mechano‐sensor and damage‐sensor in vivo

#### Apical Crumbs signalling represses Yorkie

The apical polarity determinant Crumbs was long thought to be essential for cells to maintain an apical domain, so it was surprising when loss of Crumbs was discovered to cause tissue overgrowth in *Drosophila* adult tissues, such as the wing or eye 17, 18. The overgrown *crumbs*‐mutant tissues were found to have normal apical‐basal polarity, due to the presence of the redundant factor Bazooka/Par3, and also exhibited upregulation of Yorkie‐target genes 17, 18, 24, 25, 26, 27. Crumbs was found to bind directly to Expanded, via its FERM‐binding domain, and thus to activate the canonical Hippo‐Warts kinase cascade to repress Yorkie activity 17, 18, 28, 29. Recent work has confirmed that phosphorylated Warts kinase can be detected precisely where Crumbs is localised in the developing wing 30. Thus, Crumbs is not only a key apical domain determinant, but also has a second function in activating Hippo signalling to repress Yorkie (Fig. 2).

#### Junctional Ft‐Ds cadherins and E‐cadherin associated signals regulate Yorkie

The planar polarity determinants Fat (Ft) and Dachsous (Ds) are atypical cadherins that localise to adherens junctions with E‐cadherin, but in an asymmetric fashion 31, 32 (Fig. 3). Ft‐Ds interactions are well known to cause the planar polarisation of the atypical myosin Dachs (D), which acts as an F‐actin motor protein to increase tension at adherens junctions to promote tissue elongation via biasing the orientation of cell divisions and cell‐cell rearrangements 33, 34, 35, 36 (Fig. 3). Interestingly, loss of Fat produces not only a failure of tissue elongation, but also tissue overgrowth due to activation of Yorkie‐target genes 19, 20, 21, 22, 34, 37 (Fig. 3). This activation of Yorkie‐driven growth was found to depend strictly on the accumulation of the Dachs myosin at adherens junctions, and stabilisation of Dachs at junctions is sufficient to drive tissue overgrowth 34, 38, 39. Dachs appears to activate Yorkie by antagonising Warts 40, but the kinases Minibrain (Mnb) and Riquiqui (Riq) also appear to be involved and these can directly phosphorylate Warts to repress its activity 41 (Fig. 3).

The F‐actin associated proteins Ajuba, Zyxin, and Src localise to adherens junctions and promote activation of Yorkie‐target genes and tissue growth in the fly wing and eye 23, 42, 43, 44, 45, 46, 47, 48. Since mammalian Src family kinases (Src, Fyn, Yes) are known to phosphorylate and activate mammalian homologues of Yorkie (YAP, or Yes‐associated protein, and TAZ) 49, 50, it is plausible that Ajuba and Zyxin act to promote Src activity at adherens junctions and thereby activate Yorkie to drive tissue growth. Alternatively, Ajuba and Zyxin may directly inhibit the Warts kinase, to reduce the inhibitory phosphorylation of Yorkie by this kinase 23.

#### Basal Integrin signalling may activate Yorkie in intestinal stem cells

Integrins are localised to the basal side of epithelial cells, where they play a key role in cell adhesion to the extracellular matrix 51. In the *Drosophila* intestine, proliferation of stem cells depends critically on Integrins and their intracellular signal transducers such as Talin 52, 53. How Integrin signalling promotes stem cell proliferation remains unclear, but both Src and Yorkie are of pivotal importance for proliferation of these cells, suggesting a potential regulatory connection 54. Notably, intestinal stem cells lack an apical domain, so are likely to have no Crumbs‐Hippo‐Warts signalling and thus strongly active Yorkie that requires input from basal Integrin‐Src signalling to maintain stem cell proliferation. Thus, Yorkie appears to act as a sensor of cell polarity to promote proliferation in stem cell populations.

#### Mechanical stretching activates Yorkie

In addition to acting as a sensor of cell polarity, another possible physiological function for Yorkie is as a mechanosensor – originally proposed in mammalian cell culture for YAP/TAZ 55, 56. In the developing fly wing, peripheral epithelial cells become circumferentially stretched by the morphogen‐driven growth of the central wing pouch, and the stretched cells respond by proliferating more to produce a near‐uniform level of proliferation across the entire tissue 57, 58, 59, 60, 61. How these cells sense mechanical forces was unclear, until it was revealed that the degree of stretching correlated with increased Yorkie‐target gene activity 24.

One possible mechanosensor is Crumbs itself, which binds to the apical Spectrin cytoskeleton – a mechanically deformable network that is required for Crumbs to activate Hippo signalling 24, 62. Stretching of the apical domain of the cell correlates with a decrease in the local density of Crumbs molecules, which may then decrease the ability of Crumbs to activate Hippo‐Warts signalling and repress Yorkie 24. In support of this model, either forcing the clustering of Crumbs with an extracellular ligand (Crumbs itself expressed on neighbouring cells) or increasing the local concentration of Hippo kinase can strongly activate Hippo‐Warts signalling and prevent stretch‐induced Yorkie activation 24. Interestingly, mechanical stretching of wing cells also leads to increased recruitment of phosphorylated myosin‐II 60, and the same phenomenon occurs upon disruption of the apical Spectrin cytoskeleton 62, again supporting a mechanosensory role for Spectrins.

Another possible mechanosensor is the adherens junction, whose associated proteins Ajuba and Zyxin promote Yorkie activation in response to force upon the actomyosin cytoskeleton 23, 44. It will be interesting to test whether the physiological stretch forces that occur during development are sufficient to affect recruitment of Ajuba and Zyxin and their ability to activate Src and/or inhibit Warts. Presently, there is evidence that non‐physiological reduction of acto‐myosin contractility at adherens junctions leads to reduced Ajuba association 23, but this may simply be due to the requirement for actomyosin in maintaining the adherens junctions themselves. One plausible mechanism for mechanosensing is via the alpha‐catenin protein, which can unfold under force to reveal a Vinculin binding site, but further work is necessary to test the role of Vinculin in regulation of Yorkie 63.

#### Tissue damage activates Yorkie

Another physiological function for Yorkie is sensing tissue damage 64. This role is best understood in the *Drosophila* intestine, where damaging agents such as pathogenic bacteria or chemical treatment with the insecticide Paraquat produce a massive stem cell proliferation response to regenerate the tissue that depends upon Yorkie activity 7, 8, 9. Interestingly, Yorkie is required both in the stem cells for proliferation and in the differentiated epithelial cells to sense damage. Yorkie induces expression of JAK‐STAT pathway ligands (Upds) which signal to stem cells to further promote their proliferation 7, 8, 9. Precisely, how Yorkie senses tissue damage remains unclear, and this is a fundamentally important question to answer to fully understand the physiological roles of Yorkie in vivo. Recent work suggests that Yorkie inhibits the infection‐sensing Toll receptor – Dorsal/NF‐kappaB pathway, which in turn inhibits Yorkie activation 65. Thus, tissue damage sensing by Yorkie is likely to act in a parallel and antagonistic manner to infection sensing, perhaps to promote a sterile‐inflammation response rather than an infection response. Further work is necessary to explore this possible role of Yorkie.

### YAP/TAZ as polarity sensor in vivo

#### Repression of YAP/TAZ by apical signals

The mammalian Yorkie homologs YAP and TAZ are clearly regulated by the presence or absence of an apical domain in mammalian epithelial cells 49. In columnar epithelia, YAP and TAZ remain cytoplasmic, while in basal layer epithelial stem cells that lack an apical domain, YAP and TAZ localise to the nucleus 49. The organisation of the bronchial epithelium is a good example of this phenomenon, and it has been shown that apical signalling requires CRB3, a Crumbs homolog 66 (Fig. 2).

#### Stimulation of YAP/TAZ by basal signals

Nuclear localisation of YAP and TAZ also appears to be promoted by Integrin signalling upon attachment of cells to the basement membrane 49, 67, 68. In squamous epithelia, YAP and TAZ are nuclear in basal layer stem/progenitor cells but cytoplasmic in most suprabasal differentiating cells, despite the fact that squamous epithelial cells never differentiate an apical domain 49 (Fig. 1). This finding suggests that loss of Integrin‐mediated contact with the basement membrane extracellular matrix triggers relocalisation of YAP and TAZ. The organisation of the skin epithelium is a good example of this mode of regulation, and regulation of YAP has been shown to depend on Integrin‐Src signalling in this tissue to drive proliferation of basal layer stem/progenitor cells 49, 67, 68 (Fig. 1). It will be interesting to test whether Integrin‐Src signalling regulates YAP/TAZ in other tissues where Integrins, Src or YAP are known to drive cell proliferation such as during liver regeneration 69, 70.

#### A role for signalling from adherens junctions?

Whether junctionally localised factors directly regulate YAP and TAZ remains controversial. The atypical cadherins Fat and Dachsous have multiple homologs in mammals, but knockouts tend to affect tissue shape rather than tissue growth in epithelia 71 (but they reveal a role in both neural and nephron proliferation 72, 73, 74, 75). Furthermore, disruption of adherens junctions in alpha‐catenin knockout skin does not lead to a reduction in YAP/TAZ activity or reduced cell proliferation, but rather leads to overproliferation – suggesting a possibly indirect activation of YAP/TAZ via increased Integrin‐Src signalling in alpha‐catenin knockout skin 50, 76, 77. Further work is necessary to investigate whether junctionally associated Zyxin/TRIP6, Ajuba/WTIP or Src family members contribute to YAP/TAZ activation in vivo, as they do for *Drosophila* Yorkie (Fig. 3).

### YAP/TAZ as mechano‐sensor in vivo

YAP and TAZ were first proposed to be mechanosensors based on results in cell culture 55, 56. The key regulatory mechanism in cell culture is the attachment of cells to their basal substratum via Integrins 49, 67, 68, 78, whose ‘outside‐in’ signalling is firmly implicated in mechanosensation 79, 80, 81, 82, 83, 84, 85, 86, 87, 88, 89, 90, 91 (Fig. 4). In addition, apical signals from Crumbs‐Hippo‐Warts may be reduced upon stretching of the apical Spectrin cytoskeleton 24 while adherens junction or Integrin signals via Zyxin‐Ajuba‐Vinculin‐Src‐FAK may be increased due to the actomyosin contractile response to tension or stretch 60, 92, which then induces clustering and activation of E‐cadherin or Integrins and thus Src and FAK to drive YAP/TAZ to the nucleus 93, 94, 95, 96, 97, 98, 99, 100, 101, 102, 103, 104, 105 (Fig. 4). Importantly, there is not yet conclusive evidence that YAP or TAZ (unlike Yorkie) can respond to mechanical force in vivo. Since YAP and TAZ promote normal cell proliferation in the skin, it may be that the stretching of the skin during post‐natal growth or adult obesity induce YAP and TAZ activity to enable the skin to grow to cover the entire surface area of the body. Further work is necessary to develop mechanical stretching systems for epithelia to measure the requirement for YAP and TAZ in stretch‐dependent growth in vivo.

### YAP/TAZ as damage‐sensor in vivo

In the mammalian intestinal epithelium, overexpression of YAP was found to be sufficient to promote increased stem cell proliferation 106. Notably, YAP and TAZ double conditional knockout mice appear not to affect normal gut homeostasis, but even YAP single‐knockouts reduce the tissue damage‐induced or APC‐mutant induced proliferation response of this tissue 107, 108, 109, 110. Note that there is much controversy over how APC loss leads to YAP/TAZ nuclear localisation 107, 108, 109, 110, and that YAP can in fact remain cytoplasmic in human or mouse adenomas that retain an apical domain and normal columnar organisation 49. Importantly, both tissue damage and APC loss lead to a strong increase in YAP levels, which may include increased transcription of YAP as well as stabilisation of the YAP protein 107, 108. Mechanistically, cytokine receptor signalling, particularly the gp130 co‐receptor, has been implicated in activating Src family kinases and YAP in the mouse intestine in response to mucosal injury to promote proliferative wound healing in the mouse gut 111. Independent work confirms a key role for Src kinase in mouse intestinal proliferation and tumour formation 54. Whether cytokine receptors are the sole signal regulating Src and/or YAP/TAZ in damaged tissues remains to be clarified.

In the mammalian skin epithelium, overexpression of YAP is also sufficient to promote increased basal layer proliferation 76, 77, 112. Double conditional knockouts for YAP and TAZ reduce skin proliferation and also reduce the ability of skin wounds to heal 49. As in the gut, damage‐induced upregulation of YAP and TAZ can be observed around the wound site, suggesting that these factors may directly respond to tissue damage to promote the proliferative response 49. The elevation of YAP/TAZ levels in response to skin damage requires Src family kinase signalling, although the signals acting upstream of Src remain unclear 49. It will also be interesting to test whether YAP and TAZ contribute to the inflammatory response that often accompanies different types of tissue damage.

In the mammalian liver, overexpression of YAP, or loss of upstream components of the Hippo pathway such as Merlin/Sav, MST1/2, or Mob1a/1b drive tissue overgrowth 69, 113, 114, 115, 116, 117. YAP knockout livers are relatively normal sized but lose some hepatocytes and biliary cells 113. It will be interesting to see whether the YAP/TAZ double knockout livers are also normally sized, and whether they have difficulty in regenerating after partial hepatectomy 118, 119.

### YAP/TAZ in human epithelial cancers

Most human cancers are epithelial in origin and progression towards malignant carcinoma involves a disruption of apical‐basal polarisation, invasive migration and damage/inflammation. All three of these malignant changes would be expected to induce YAP/TAZ nuclear localisation. Loss of the apical domain would be predicted to disrupt Crumbs‐Hippo signalling to activate YAP/TAZ 49, 66, 68. Increased contact with the extracellular matrix would be predicted to increase Integrin‐Src signalling to activate YAP/TAZ 49, 67, 68. Invasive migration involves force‐generation that may further activate Integrin‐Src signalling and YAP/TAZ 49, 67, 68. Damage and/or inflammatory responses may also contribute to stimulation of YAP/TAZ activity in cancer 49, 54, 68, 111. Notably, these responses may not be limited only to the proliferating cancer cells themselves but also occur in the cancer‐associated fibroblasts that promote tumour invasion 78. Thus, YAP/TAZ may be the missing link that explains why cancers appear to behave as ‘the wound that never heals’, why inflammation promotes malignancy or why disruption of epithelial polarity and morphology is such a universal and predictive hallmark of malignant carcinomas.

### Are other functions proposed for YAP/TAZ in vitro operative in vivo?

Since the localisation and activity of YAP and TAZ can be readily examined in cell culture, a veritable myriad of interventions have been proposed to affect their activity in these assays. For the most part, there is no evidence that any of these cell culture discoveries actually reflect a physiologically relevant mechanism of YAP/TAZ regulation in vivo. Here we focus on just a few examples.

#### Does Wnt signalling activate YAP/TAZ, or vice versa, in vivo?

YAP/TAZ was reported to inhibit Wnt signalling via interactions between YAP/TAZ and either beta‐catenin 120, dishevelled 121, or the axin/beta‐TrCP destruction complex 109. These reports predict that loss of YAP/TAZ should result in activation of Wnt‐beta‐catenin signalling in vivo, and this does not appear to be the case in the YAP/TAZ double knockout mouse intestine 108, 109 or in *Drosophila yorkie* or *mask* mutants or RNAi 6, 10, 29. Furthermore, these reports also predict that overexpression of YAP or Yorkie should inhibit Wnt‐beta‐catenin signalling in vivo, and once again there is no convincing evidence for this effect in mice or flies 6, 10, 29.

More plausible is the notion that YAP/TAZ‐TEAD and beta‐catenin‐TCF complexes may cooperate, or antagonise, on the promoters of particular target genes 122, 123. Promoters are indeed where most cross‐talk between signalling pathways to the nucleus takes place in multicellular organisms, because this mechanism allows for the combinatorial regulation of gene expression necessary for multicellular development 124. It is also plausible that Wnt‐beta‐catenin signalling may simply transcriptionally induce YAP in certain tissues such as the intestinal crypt.

A more recent report proposed that Wnt signalling activates YAP/TAZ via the non‐canonical ‘alternative’ Wnt‐Frizzled pathway 125. The Frizzled receptor family is conserved between *Drosophila* and mammals, yet in *Drosophila* loss of Frizzled signalling causes defects in planar cell polarity and/or beta‐catenin activation but not widespread tissue undergrowth or loss of Yorkie activity 126, 127. Instead, it seems that strong activation of Frizzled in cell culture can artefactually induce Rho GTPase activation and acto‐myosin contractility, which then indirectly activates YAP/TAZ, possibly via mechanical force effects 125. Analysis of Frizzled knockout mice is necessary to determine whether YAP/TAZ activation is physiologically involved in Frizzled signalling in mammals.

#### Does BMP/Smad signalling activate YAP/TAZ, or vice versa, in vivo?

YAP was initially reported to bind to the inhibitory Smad7 to antagonise Smad3/4 signalling 128. Later work proposed that YAP cooperates with Smad1 in nuclear transcription 129 and that TAZ promotes nuclear localisation and transcriptional activity of Smad2/3‐4 complexes 130. Next, YAP/TAZ was proposed to bind to Smad2/3 to retain them in the cytoplasm in cells cultured at high density, such that both signal transducers become nuclear at low density 131. This latter work was challenged by a recent report that cell density regulates Smad activation via its effects on the subcellular localisation of TGF‐beta/BMP receptors, rather than via YAP/TAZ 132, and forced a response from the first group 133. An independent group reported that the interaction of YAP/TAZ with Smad2/3 was cell‐type specific 134. Notably, the BMP/Smad (*Drosophila* Dpp/Mad) pathway is conserved in *Drosophila* but genetic analysis has revealed no evidence of direct crosstalk with Hippo‐Yorkie signalling (although there may be indirect effects via Ds‐Ft‐Fj gradients) 34. Further work is necessary to test whether the TGFbeta/Smad2 (*Drosophila* Activin/Smad2) pathway might affect Yorkie in *Drosophila* 135. It will also be interesting to genetically test whether crosstalk between YAP/TAZ and Smad signalling operates in mouse tissues.

#### Does GPCR signalling activate YAP/TAZ in vivo?

The G‐protein‐coupled receptor (GPCR) agonist ligands lysophosphatidic acid (LPA) and sphingosine 1‐phosphate (S1P) were reported to activate YAP/TAZ via the G_12/13_ or G_q/11_ protein in cell culture, which activates Rho GTPase to alter actomyosin contractility 136, 137, 138. Other GPCR agonist ligands glucagon and epinephrine were found to inhibit YAP/TAZ activity via G_S_, cAMP and protein kinase A (PKA) 136, 139. Whether any of these signals physiologically regulate YAP/TAZ in vivo remains unclear. The only apparently supporting evidence from *Drosophila* is that *pka* mutant tissue overproliferates (though this may be due to ectopic Hedgehog signalling), while overexpression of PKA causes apoptosis 139. On the contrary, the effect of *pka* silencing by RNAi on Yorkie target genes *cyclinE* or *expanded* is very mild 139. Further work is needed to clarify whether GPCRs or PKA are truly physiologically involved in regulation of Yorkie or YAP/TAZ in vivo.

#### Does the Mevalonate pathway activate YAP/TAZ in vivo?

Two reports suggested that YAP/TAZ nuclear localisation was dependent on the SREBP/Mevalonate pathway, which turns acetyl‐CoA via mevalonate into lipid precursors such as Farnesyl‐PP (a cholesterol and other sterol precursor) and GeranylGeranyl‐PP (a precursor for prenylation of proteins such as small GTPases) 140, 141. The proposed mechanism was that inhibition of mevalonate biosynthesis by Statins (which inhibit HMG‐CoA reductase) impairs prenylation of the Rho GTPase. However, patients taking Statin drugs do not report massive side effects on stem cell proliferation and tissue homeostasis, nor are Statins known to have potent anti‐cancer effects in many solid tumour types, suggesting that Statins cannot completely inhibit the action of YAP and TAZ in vivo. In addition, the SREBP/Mevalonate pathway is conserved in *Drosophila* but appears to specifically affect lipid synthesis and cell growth rather than produce Yorkie‐like proliferation phenotypes 142, 143. Finally, there is no evidence that regulation of Rho GTPase prenylation is a physiological mechanism of YAP/TAZ regulation in vivo.

#### Do growth factors such as EGF receptor ligands or other RTK ligands activate YAP/TAZ in vivo?

The receptor tyrosine kinase (RTK) family of plasma membrane receptors is defined by a variable extracellular domain and a common intracellular tyrosine kinase domain and includes EGFRs, InsulinR, IGF1R, PDGFRs, CSF1R, Kit, Flk2, FGFRs, TrkA/B/C, AXL, Ret, ALK, DDR1/2, Ros and Eph receptors. Binding of ligands such as EGF to the EGFR leads to Tyrosine Kinase activation and trans‐phosphorylation, which then recruits signal transducers to the multiple phospho‐Tyrosine motifs in the intracellular domain. The most commonly activated signal transduction pathways downstream of RTK activation include Ras‐MAPK, Src family kinase, PI3K‐Akt‐TOR, PLCgamma, and Vav signalling – with different RTKs activating specific subsets of these pathways. In cell culture, it was reported that addition of EGF to cells was able to induce nuclear localisation of YAP/TAZ in a PI3K‐dependent fashion 67, 144. Results in *Drosophila* support the notion that PI3K signalling can activate Yorkie 145. There is also a requirement for minimal TOR activation to maintain Yorkie activity in *Drosophila* 146. In mice, there is a good correlation between EGF ligand and receptor expression and YAP/TAZ nuclear localisation in skin 49, and EGF ligands such as amphiregulin (AREG) are also transcriptional targets of YAP/TAZ in several tissues, forming a possible positive feedback loop in vivo 74, 110, 147, 148. Overall, these promising results support the notion that certain forms of RTK signalling might physiologically regulate YAP/TAZ activity in vivo. Further work in vertebrate models will be necessary to establish which RTKs are genetically required to regulate YAP/TAZ in different tissues, and how RTKs might cross‐talk with Integrin‐Src or E‐cadherin‐Src signals in vivo.

## Conclusions

Results from *Drosophila* and mouse genetics firmly establish the Yorkie/YAP/TAZ family as a sensor of cell polarity, mechanical forces and tissue damage in vivo. These three inputs are frequently misregulated in cancer, providing a possible explanation for the frequent nuclear localisation of YAP/TAZ in malignant tumour cells, where YAP/TAZ appear to contribute to malignant behaviour. Future work will need to examine the mechanism by which YAP/TAZ can respond to these physiological signals in both normal tissues and cancers. Although other signals have been proposed to regulate YAP/TAZ in cultured cells, it remains unclear whether any of these alternative signals are truly of physiological relevance in both flies and mice. Perhaps the most promising of the newly proposed signals are the RTKs, although which of these receptors is necessary to regulate YAP/TAZ in mouse tissues or tumours requires further genetic analysis. Overall, the prospects remain bright for a crucial role for YAP/TAZ signalling in both normal tissue homeostasis and cancer, making this pathway an attractive biomarker and target for therapy.

The authors have declared no conflicts of interest.
